# Association between cumulative cisplatin dose and reproductive and sexual functions in patients with malignant ovarian germ cell tumors treated with bleomycin, etoposide, and cisplatin therapy: a case series study

**DOI:** 10.1186/s40780-022-00265-8

**Published:** 2022-11-17

**Authors:** Miki Akasaka, Toshinori Hirai, Kenta Yoshida, Eiji Kondo, Tomoaki Ikeda, Takuya Iwamoto

**Affiliations:** 1 Department of Pharmacy, Mie University Hospital, 2-174, Edobashi, Tsu, Mie 514-8507 Japan; 2 Department of Obsterics and Gynecology, Mie University Hospital, 2-174, Edobashi, Tsu, Mie 514-8507 Japan

**Keywords:** Fertility, Cancer, Cisplatin, Malignant ovarian germ cell tumor

## Abstract

**Background:**

The impact of cumulative dose of cisplatin on gonadal function has not been clarified. We evaluated whether the cumulative cisplatin dose affects the resumption of menses in patients treated with bleomycin, etoposide, and cisplatin (BEP).

**Main body:**

A case series study of women < 40 years with malignant ovarian germ cell tumors receiving BEP was conducted at Mie University Hospital. Using linear regression analysis, the correlation between the cumulative dose and resumption of menses was determined. Additionally, we compared the resumption of menses stratified by age (age < 20 years or ≥ 20 years). Ten women (median age: 20 [interquartile range: 15–26] years) have received a median of 4 cycles of BEP. The median period of resumption of menses was 5 months, which had no correlation with cumulative doses of bleomycin (143 mg/m^2^ [71–220], y = -0.0069 x + 6.15, *r* = 0.19, *P* = 0.60), etoposide (1,533 mg/m^2^ [900–2,000], y = 0.0004 x + 4.56, *r* = 0.08, *P* = 0.82), and cisplatin (363 mg/m^2^ [225–400], y = 0.01 x + 1.67, *r* = 0.35, *P* = 0.32). Although the resumption of menses was comparable across ages, the cumulative doses of cisplatin were higher in patients aged < 20 years than in those aged ≥ 20 years (400 mg/m^2^ [363–450] vs. 225 mg/m^2^ [225–350], *P* = 0.02). Similarly, patients aged < 20 years had a higher cumulative etoposide dose than those aged ≥ 20 years (2,000 mg/m^2^ [1,533–2,250] vs. 900 mg/m^2^[900–1,600], *P* = 0.03). Moreover, patients aged < 20 years received more cycles of BEP than those aged ≥ 20 years (4 cycles vs. 3 cycles, *P* = 0.03).

**Short conclusion:**

All patients can recover menses after BEP, and the resumption of menses appeared at the median period of 5 months after BEP. The timing of menses resumption did not correlate with the cumulative doses of cisplatin.

**Supplementary Information:**

The online version contains supplementary material available at 10.1186/s40780-022-00265-8.

## Background

Malignant ovarian germ cell tumors (MOGCTs) are rare cancers that typically occur in young women [1]. Cisplatin-based chemotherapy, such as bleomycin, etoposide, and cisplatin (BEP), is an established regimen for MOGCTs, and with this treatment, more than 90% of patients can survive for 5 years [2–4]. Previous studies have shown that more than 80% of women restarted regular menstruation following chemotherapy including BEP for MOGCTs [5, 6].

There was no dose–response between the likelihood of pregnancy and cisplatin dose in young women with cancers using a large dataset [7]. Guidelines have suggested that the gonadal toxicity of cisplatin varies according to the cisplatin dose in men [8–10]. Conversely, the European Society for Medical Oncology (ESMO) has reported that BEP or EP for women aged < 30 years is in the low-risk category (estimated risk is < 20%) of amenorrhea regardless of cisplatin dose, although this risk classification has not been established [10]. Therefore, it remains unclear about the dose–response association between cisplatin and gonadal toxicity before pregnancy. Since it was known that high rate of resumption of menses in patients with MOGCTs after chemotherapy, this study evaluated the timing of resumption of menses following BEP in addition to the rate of menstrual resumption in order to provide new information. This study also aimed to evaluate the association between cisplatin dose and gonadal function in female patients receiving BEP.

## Main text

### Patient data

A case series study was conducted at Mie University Hospital between January 2010 and May 2019. Eligibility criteria included patients (aged 10–40 years) who had a histological diagnosis of MOGCT. All patients underwent fertility-sparing surgeries. The exclusion criteria were as follows: (1) no record of menstrual history and (2) bilateral adnexectomy because of the difficulty of pregnancy. The study protocol was approved by the Institutional Review Board of Mie University Hospital (approval number: H2020-016). Clinical characteristics were obtained from electrical medical charts.

Throughout the diagnostic process, gynecologic pathologists diagnosed the histopathological category and classified it as MOGCT according to the World Health Organization classification criteria [11]. The clinical staging of ovarian cancer was determined based on the guidelines of the International Federation of Gynecology and Obstetrics (FIGO) 1988 from 2010 to 2014 [12] and FIGO 2014 from 2015 to 2019 [13]. In the following treatment step, all patients received BEP, which consists of bleomycin 30 units intravenously per week, etoposide 100 mg/m^2^ intravenously (on days 1–5), and cisplatin 20 mg/m^2^ intravenously (on days 1–5), lasted 21 days with 3–4 cycles as the standard schedule [8]. Furthermore, we included patients who received additional chemotherapy, vincristine, actinomycin D, and cyclophosphamide (VAC) because it remained unclear about the influence of additional cyclophosphamide on the resumption of menses in patients who received BEP. Cyclophosphamide is also reported to be at high risk of ovarian dysfunction [6].

The study outcome was defined as the period from the end of BEP to the resumption of menses. The obstetrician and gynecologist confirmed the resumption of menses during the medical interview.

### Statistical analyses

Statistical analyses were performed using JMP® Pro 16 (SAS Institute Inc., Cary, NC, USA). Non-normal distributed continuous data are expressed as median (interquartile range [IQR]) and compared using the Mann–Whitney U test. Categorical data are expressed as numbers (%). A two-tailed *P* value < 0.05 was considered statistically significant. Linear regression analysis evaluated correlations between cumulative doses and outcome. Furthermore, primordial follicle counts decrease with advancing age [14]; therefore, we compared the relative dose intensity (RDI) and outcome by age groups (< 20 years and ≥ 20 years).

## Results

Ten patients with MOGCT met the eligibility criteria. All patients underwent fertility-preserving surgical resection, and no recurrence of MOGCT was observed. The clinical characteristics are summarized in Table 1. The median age at the time of diagnosis was 20 years [15–26]. One patient was diagnosed with ovarian cancer during pregnancy, and the other patients were nullipara. Of the four patients who were married, there was only one patient who did not desire a child. In contrast, the others gave birth after the completion of chemotherapy.

**Table 1:** Bleomycin, etoposide, and cisplatin (BEP) therapy and pregnancy status in fertility-preserved patients

	Age, y	Histology type/FIGO stage	BSA, m^2^	Cumulative doses, mg/m^2^			Treatment cycle	Resumption of menses, M	Marital status	Live births
				BLM	VP-16	CDDP				
1	22	Immature teratoma/Ic	1.53	75	900	225	3	3	Yes	1
2	25	Immature teratoma/Ic	1.37	25	900	225	3	5	Yes	1
3	25	Mixed germ cell tumor/IIIb	1.53	100	1,200	300	4	6	Yes	2
4	16	Dysgerminoma/IIIc	1.57	185	1,625	325	6	2	No	-
5	29	Yolk sac tumor/Ic	1.66	75	900	225	3	3	No	-
6	14	Yolk sac tumor/Ic	1.42	210	2,500	500	5	4	No	-
7	18	Immature teratoma/Ia	1.49	220	2,000	400	4	5	No	-
8	27	Yolk sac tumor/IIIc	1.38	220	2,000	400	4	5	Yes	-
9	15	Immature teratoma/Ia	1.39	220	2,000	400	4	7	No	-
10	10	Immature teratoma/IIIc	1.24	60	1,440	400	4	12	No	-
	20 (15–26)	-	1.5 (1.4–1.5)	143 (71–220)	1,533 (900–2,000)	363 (225–400)	4 (3–4)	5 (3–6)	-	-

Continuous data are expressed as median (interquartile range)

Live births are expressed as the number after BEP therapy

Patient No. 10 received a total of 1 cycle of vinblastine, actinomycin D, and cyclophosphamide therapy following BEP therapy. Patient No. 8 had a history of pregnancy before BEP therapy, and she had no desire for additional childbearing after 4 cycles of BEP therapy

*y* Year, *M* Months, *BSA* Body surface area, *FIGO* International Federation of Gynecology and Obstetrics, *BLM* Bleomycin, *VP-16* Etoposide, *CDDP* Cisplatin

In all patients, it took a median period of 5 months to recover menses after chemotherapy. Notably, one patient (No. 10) received an additional cycle of VAC regimen following BEP. In the VAC, doses of vinblastine, actinomycin D, and cyclophosphamide were 9.4 mg, 2 mg, and 1,100 mg, respectively. One patient (No 2.) was suspected of eruption due to bleomycin during the first cycle. Therefore, after the second cycle, the patient received etoposide and cisplatin, except for bleomycin. Two cases (No. 7 and No. 8) discontinued bleomycin due to febrile neutropenia and herpes zoster in the first cycle (day 15), and bleomycin was restarted from the second cycle. Additionally, one patient (No. 8) had an ovarian tumor during pregnancy, and she gave birth to a male infant via cesarean section at 28 weeks of gestation. She had no desire for additional childbearing after four cycles of BEP.

**Fig. 1:**
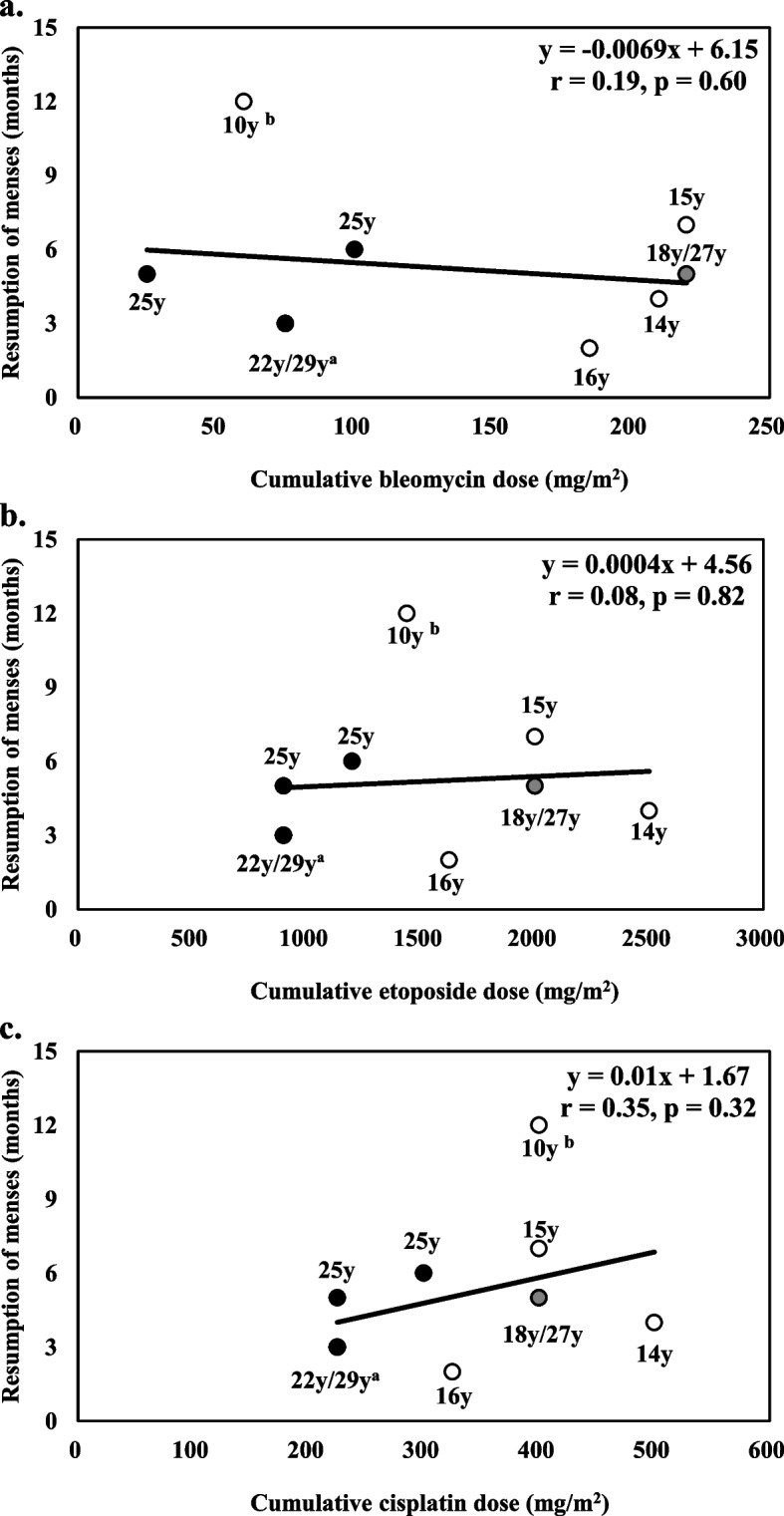
Association between cumulative doses of anticancer drugs and timing of resumption of menses. The X-axis and Y-axis represent the cumulative bleomycin (**a**), etoposide (**b**), and cisplatin (**c**) dose and the timing of resumption of menses, respectively. Open circles represent age < 20 years. Closed circles represent age ≥ 20 years. Gray points represent duplicated points (age 18 and 27 years). a: This point is duplicated in two patients (aged 22 years and 29 years). b: This point corresponds to a patient treated with vinblastine, actinomycin D, and cyclophosphamide (VAC) in addition to bleomycin, etoposide, and cisplatin (BEP)

The cumulative doses for BEP did not correlate with outcome (Fig. 1A, *r* = 0.19, *P* = 0.60; Fig. 1B, *r* = 0.08, *P* = 0.82; Fig. 1C, *r* = 0.35, *P* = 0.32). Additionally, there was no significant difference in the timing of resumption of menses between patients aged < 20 years and ≥ 20 years (5 months [3–10] vs. 5 months [3–6], *P* = 0.40). Conversely, patients aged < 20 years received more cycles of BEP than those aged ≥ 20 years (4 cycles [4–6] vs. 3 cycles [3, 4], *P* = 0.03). There was no difference in RDI (%) for each drug across age groups (Additional file 1).

## Discussion

Our study revealed that all patients recovered, and the resumption of menses appeared at a median period of 5 months after BEP. Additionally, three of the four married patients gave birth after the completion of BEP. These results are supported by the statement that BEP for female patients aged < 30 years is in the low-risk category of amenorrhea regardless of cisplatin dose [10]. Tamauchi et al. also showed that 42 of the 45 MOGCT survivors became pregnant and 40 successfully gave birth. Of these 40 patients, 50% had received BEP, however the cumulative dose of anticancer drugs and the timing of menstrual resumption were unknown [15]. In previous reports, more than 80% of the patients resumed menstruation following cisplatin-based chemotherapy including BEP for MOGCTs [5, 6], which discord with our findings. Conversely, Pektasides et al. showed patients largely restarted the resumption of menses under POMB/ACE regimen (cisplatin, vincristine, methotrexate, bleomycin/actinomycin D, cyclophosphamide, etoposide) with the resumption of menses of 4.5 months after chemotherapy [16], which is consistent with our findings. This difference might be attributed to details of chemotherapy although information from previous studies is lacking.

Our result revealed no correlation between cumulative cisplatin dose and resumption of menses under standard treatment cycles. Satoh et al. reported that most patients aged < 40 years who received BEP at 20 mg/m^2^/day of cisplatin on days 1–5 recovered menstruation [17]. Thus, the standard treatment intensity of cisplatin can maintain reproductive function in patients with MOGCT. In our study, no effect of age on the timing of menstrual resumption was identified. On the other hand, it was observed that the patients aged < 20 years received more intensified BEP than those aged ≥ 20 years. The ovarian function is relatively high in young patients [15]. Accordingly, young patients can tolerate gonadal toxicity against the intensified BEP. There was an insignificant difference in the resumption of menses comparing patients < 20 years to those ≥ 20 years, suggesting that younger age groups have better tolerance for gonadal toxicity. The association between cumulative dose of cisplatin and the timing of resumption of menses in under 30 years old should be future topic to be considered.

One patient (No. 10) experienced relatively long-term amenorrhea due to cyclophosphamide, corresponded to a high risk of amenorrhea. Notably, she received BEP with a similar intensity compared to the other patients. The ESMO guidelines suggest that even in Ewing’s sarcoma, female patients (< 35 years) had a low-risk of amenorrhea against multi-agent chemotherapy including cyclophosphamide [10]. However, cyclophosphamide certainly decreases gonadal function [8]. Similarly, a single cycle of cyclophosphamide (890 mg/m^2^) prolonged the resumption of menses in the patient. Close attention should be paid to the cyclophosphamide-based regimen.

This study has some limitations in interpreting the results. First, this study included a small number of patients. Second, this study was a retrospective design, we could not confirm the causality of chemotherapy and amenorrhea in detail. Third, the study patients aged < 30 years, and our results cannot be extrapolated to older patients who desire childbearing. Fourth, we did not collect the serum levels of anti-Müllerian hormone, a surrogate marker of sexual function [18].

## Conclusions

In summary, all patients with a standard intensity of BEP were able to resume menses at a median of 5 months.　The timing of menses resumption did not correlate with the cumulative doses of cisplatin.　This information will be useful in educating patients prior to starting chemotherapy.

## Supplementary Information

**Additional file 1.** Relative dose intensity of BEP and resumption of menses in the two groups divided by age.

## Data Availability

All the data generated or analyzed in this study are included in the published article.

## Abbreviations

BEP: Bleomycin, etoposide, and cisplatin
MOGCTs: Malignant ovarian germ cell tumors
ESMO: The European Society for Medical Oncology
FIGO: The International Federation of Gynecology and Obstetrics
VAC: Vincristine, actinomycin D, and cyclophosphamide
RDI: Relative dose intensity
