# Supplementary material for: Association between cumulative cisplatin dose and reproductive and sexual functions in patients with malignant ovarian germ cell tumors treated with bleomycin, etoposide, and cisplatin therapy: a case series study
Source: J Pharm Health Care Sci. 2022 Nov 17;8:33. doi: 10.1186/s40780-022-00265-8 (PMC9673296; doi:10.1186/s40780-022-00265-8)
Supplement: Supplementary file 1 — Additional file 1. Relative dose intensity of BEP and resumption of menses in the two groups divided by age. [file 40780_2022_265_MOESM1_ESM.docx]

**Additional file**

**Additional file 1** Relative dose intensity of BEP and resumption of menses in the two groups divided by age

Continuous data are expressed as median [interquartile range] and compared using the Mann–Whitney U test.

Relative dose intensity (%) = actual dose (mg) ×100/planned dose (mg).

BLM, bleomycin; VP-16, etoposide; CDDP, cisplatin.

P-values were tested for <20years and ≥20years.

|  | **Overall (n = 10)** | **<20 years (n = 5)** | **≥20 years (n = 5)** | **P** |
| --- | --- | --- | --- | --- |
| **BLM** |  |  |  |  |
| Cumulative doses (mg/m^2^) | 143 [71–220] | 210 [123–220] | 75 [50–160] | 0.24 |
| Relative dose intensity (%) | 98.2 [91.5–101.1] | 98.6 [83.3–104.3] | 97.8 [91.5–101.1] | 0.75 |
| **VP-16** |  |  |  |  |
| Cumulative doses (mg/m^2^) | 1,533 [900–2,000] | 2,000 [1,533–2,250] | 900 [900–1,600] | 0.04 |
| Relative dose intensity (%) | 98.6 [96.4–99.5] | 98.6 [84.3–99.1] | 98.5 [94.8–100.0] | 0.60 |
| **CDDP** |  |  |  |  |
| Cumulative doses (mg/m^2^) | 363 [225–400] | 400 [363–450] | 225 [225–350] | 0.03 |
| Relative dose intensity (%) | 97.2 [95.9–98.4] | 97.1 [84.8–98.5] | 97.3 [95.9–98.9] | 0.92 |
| **Resumption of menses (months)** | 5 [3.0–6.3] | 5 [3.0–9.5] | 5 [3.0–9.5] | 0.60 |
